# Reversible Covalent PROTACs: Novel and Efficient Targeted Degradation Strategy

**DOI:** 10.3389/fchem.2021.691093

**Published:** 2021-07-05

**Authors:** Minghua Yuan, Yanan Chu, Yongtao Duan

**Affiliations:** ^1^ Henan Provincial Key Laboratory of Children’s Genetics and Metabolic Diseases, Children’s Hospital Affiliated to Zhengzhou University, Henan Children’s Hospital, Zhengzhou Children’s Hospital, Zhengzhou, China; ^2^ School of Pharmaceutical Science, Zhengzhou University, Zhengzhou, China

**Keywords:** reversible covalent, PROTACs, degradation, drug design, selectivity, catalysis

## Abstract

The proteolysis targeting chimeras (PROTACs), which are composed of a target protein binding moiety, a linker, and an E3 ubiquitin ligase binder, have been a promising strategy for drug design and discovery. Given the advantages of potency, selectivity, and drug resistance over inhibitors, several PROTACs have been reported in literature, which mostly focus on noncovalent or irreversible covalent binding to the target proteins. However, it must be noted that noncovalent or irreversible PROTACs have several drawbacks such as weak binding affinity and unpredictable off-target effects. Reversible covalent PROTACs, with properties of enhanced potency, selectivity, and long duration of action, have attracted an increasing amount of attention. Here, we propose a comparison between these three patterns and highlight that reversible covalent PROTACs could pave the way for a wide variety of challenging target degradations.

Recently, proteolysis targeting chimeras (PROTACs) have been an exciting strategy for modulating a protein of interest by degradation, which was first reported by Crew and Deshaies in 2001 (Sakamoto et al., 2001). It is a bifunctional molecule consisting of three parts: One end is the ligand that binds to the target protein, one end is another ligand that binds to the E3 ubiquitin ligase, and the middle section is the linker (Gadd et al., 2017a). PROTACs recruit a non-native target protein into the proximity of the E3 ligase so that the target protein can be labeled with ubiquitination, which leads to degradation induced by the ubiquitin–proteasome system (UPS) (Riching et al., 2018). This drug design strategy has increasingly attracted attention, especially upon the first PROTAC entering clinical trials in 2019 (Mullard, 2019).

Even though PROTACs have very large molecular weights, poor permeability, and lack of rational optimization strategies, they still have many advantages, such as defined degradation mechanisms (Riching et al., 2018; Bhatt et al., 2019; Xia et al., 2019) and facile modular design (Gadd et al., 2017b). For degradation, PROTACs must bind target proteins and E3 ubiquitin ligases. However, many targets such as transcription factors (Brennan et al., 2008; Koehler, 2010) are recalcitrant to ligand discovery, and efficient recruiters are popular for only a handful of E3 ligases such as CRBN (Lu et al., 2015), VHL (Gadd et al., 2017a), IAP (Naito et al., 2019), and MDM2 (Hines et al., 2018). This review introduces binding patterns of E3 ligases consisting of irreversible covalent, reversible noncovalent, and reversible covalent binding. Irreversible covalent binding to E3 ligases can recruit multiple target molecules for ubiquitination and degradation without the need for the kinetic process of forming the E3-PROTAC complexes (Gabizon and London, 2021), which is shown in Figure 1(black). As a possible mechanism of action, reversible covalent binding offers the potential for sustained target engagement and avoids permanent protein modification (Tong et al., 2020).

**FIGURE 1:**
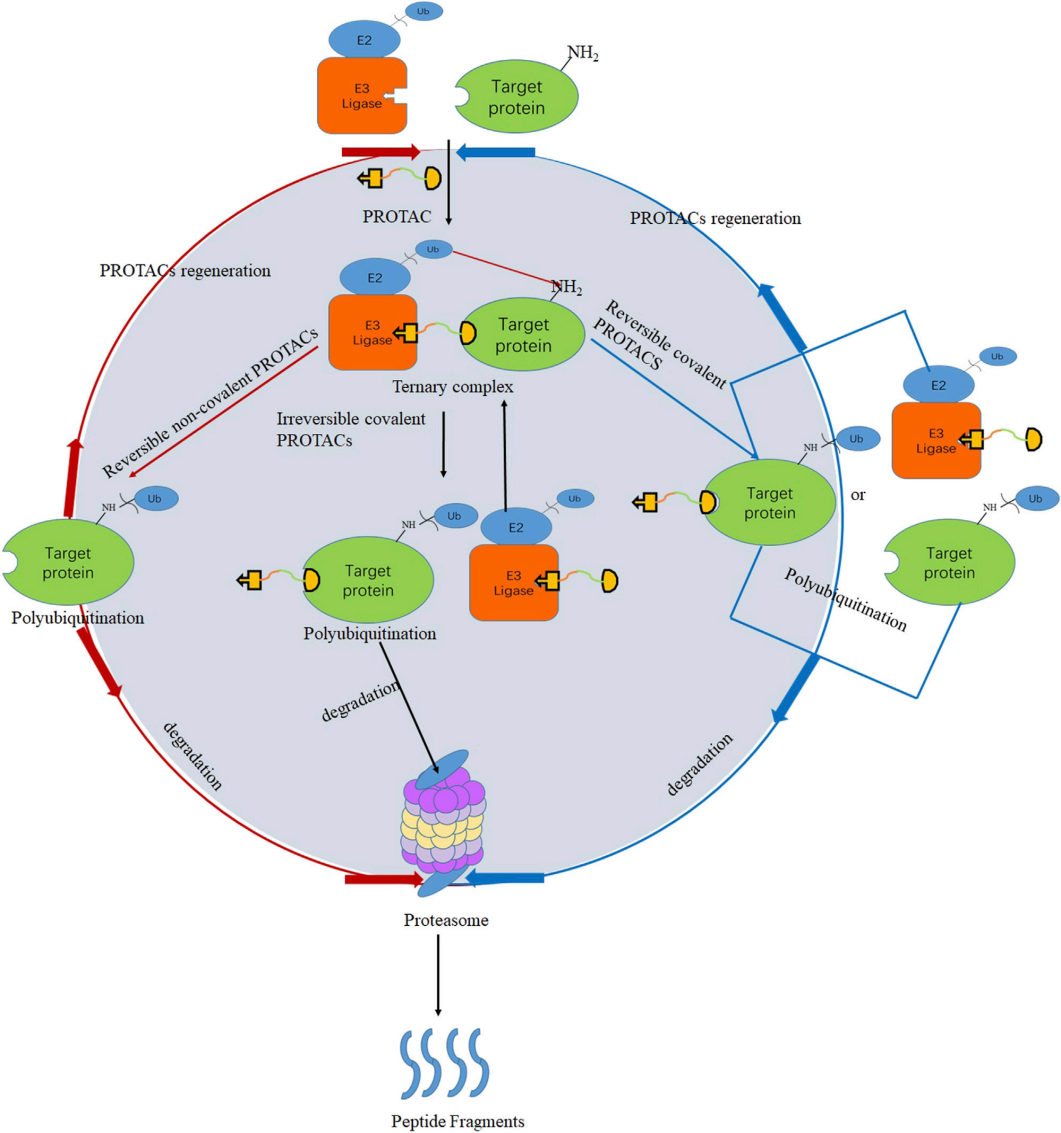
PROTACs’ mechanism for irreversible covalent PROTACs (black), reversible covalent PROTACs (blue), and reversible noncovalent PROTACs (red).

Currently, most reported PROTACs bind to the target proteins by the means of reversible noncovalent pattern, and different kinds of proteins have been successfully degraded by this strategy, such as TANK-binding kinase 1 (TBK1) (Crew et al., 2017) and cyclin-dependent kinase 9 (CDK9) (Olson et al., 2017). Many potent and selective hydroxyproline-based PROTACs have been recently reported against a wide range of target proteins, including bromodomain-containing protein 4 (BRD4) (Testa et al., 2018) and receptor-interacting serine-threonine kinase 2 (RIPK2) (Bondeson et al., 2015). However, some researchers reported that reversible noncovalent PROTACs have poor selectivity. Remillard connected the BRD4 inhibitor JQ1 and CRBN ligand to design a PROTAC that could simultaneously degrade multiple proteins of the BRD family, including BRD2, BRD3, and BRD4 (Lu et al., 2015). Research from the Bondeson group used foretinib as the target protein binding part and VHL as the E3 ubiquitin ligase ligand, respectively, to design a PROTAC that can degrade a total of nine kinases simultaneously (Bondeson et al., 2017). An explanation is that reversible noncovalent PROTACs could recruit multiple proteins and E3 ligases and then form ternary complexes to make protein ubiquitination and degradation. Due to the strong affinity and potent occupancy ability, irreversible covalent PROTACs have also successfully degraded target proteins such as HaloTag-fused cAMP-responsive element-binding protein 1 (HaloTag-CREB1), HaloTag-fused c-jun (HaloTag-c-jun) (Tomoshige et al., 2016), recombinant methionyl aminopeptidase 2 (MetAP-2) (Sakamoto et al., 2001), and Bruton’s tyrosine kinase (BTK) (Xue et al., 2020). Nevertheless, as shown in Figure 1, once the irreversible covalent PROTACs form a ternary complex with the target protein and E3 ubiquitin ligase, they will be directly degraded by the proteasome and cannot be recycled. Undoubtedly, irreversible covalent binding may reduce potency by negating the catalytic nature of the PROTAC’s activity (Bondeson et al., 2015; Lebraud et al., 2016). Furthermore, some studies reported that irreversible covalent PROTACs inhibited the degradation of target proteins and even irreversibly bound to other biomolecules to cause off-target toxicity (Dahal et al., 2013; Burslem et al., 2017; Tinworth et al., 2019).

Reversible covalent PROTACs are theorized to combine the benefits of covalent bond formation with the substoichiometric target turnover achieved by reversible PROTACs, which is unattainable for covalent PROTACs (excluding PROTACs with covalent ligands for the E3 ligase) (KielyCollins et al., 2021). Compared with the other two types of PROTACs, reversible covalent PROTACs have better target selectivity and lower potential toxicity (Gabizon et al., 2020a; Guo W. H. et al., 2020; KielyCollins et al., 2021). As a possible mechanism of action, which is described in Figure 1 (blue), *in vitro*/*vivo*, the ligand parts of reversible covalent PROTACs bind to the target proteins or E3 ubiquitin ligases through reversible covalent bonds, thereby forming stable ternary complexes. Ubiquitin located on the E2 ubiquitin-conjugating enzymes is then transferred to the target protein, which leads to the ubiquitination of the target proteins and degradation by the proteasome. PROTACs are released from the target protein or the E3 ligase, and a new ternary complex is formed again.

As far as known to the authors, researches in this area are scarce. We have listed some reported reversible covalent PROTACs, as shown in Figure 2. Maimone and his cooperators were the first to design a reversible covalent PROTAC CDDO-JQ1 based on bardoxolone, which successfully degraded BRD4. The reversible covalent moiety of CDDO-JQ1 was the E3 ligase recruiter (Tong et al.; Tong et al., 2020). Following this, Jin Wang and Nir London designed reversible covalent PROTACs, which both used the first FDA-approved covalent kinase inhibitor ibrutinib as the target protein moiety and chose pomalidomide as the CRBN E3 ligase binder, successfully degrading Bruton’s tyrosine kinase (BTK). Most PROTACs have poor cell uptake capacity and membrane permeability due to their large molecular weight. The Jin Wang research group certified through SPPIER imaging that reversible covalent PROTAC RC-1 is more efficient in inducing BTK-PROTAC-CRBN ternary complexes formation in living cells compared to the other two types PROTACs. They enhanced the accumulation of PROTACs in cells and their binding ability by introducing reversible covalent groups. Inspired by Jin Wang’s idea, another target protein (Fms-like tyrosine kinase 3) had been degraded by this same strategy (Guo W. H. et al., 2020). The Nir London group’s research proved that reversible covalent PROTACs based on dimethylated cyanoacrylamide could form covalent complexes more rapidly and validated reversible binding by the ibrutinib competition assay. Moreover, they made a comparison among these three types and found that only the reversible covalent PROTAC RC-3 degraded a known ibrutinib off-target BLK (a covalent off-target of ibrutinib) with no activity against the noncovalent off-targets CSK and LYN, representing enhanced selectivity. In essence, their research suggested that degradation by reversible covalent PROTACs was driven by covalent engagement and exhibited enhanced selectivity toward BTK compared to noncovalent and irreversible covalent PROTACs (Gabizon et al., 2020b). The aforementioned PROTACs may form reversible covalent complexes with the target proteins or E3 ubiquitin ligases. The PROTACs are then released and form a ternary complex again, thus inducing protein degradation in a substoichiometric/catalytic manner. The advantages of reversible covalent PROTACs are evident in Table 1. Furthermore, many reports had proven that covalent enzyme inhibitors displayed powerful therapeutics and exquisite selectivity by using reversible covalent warheads in drug design and discovery (Bandyopadhyay and Gao, 2016).

**FIGURE 2:**
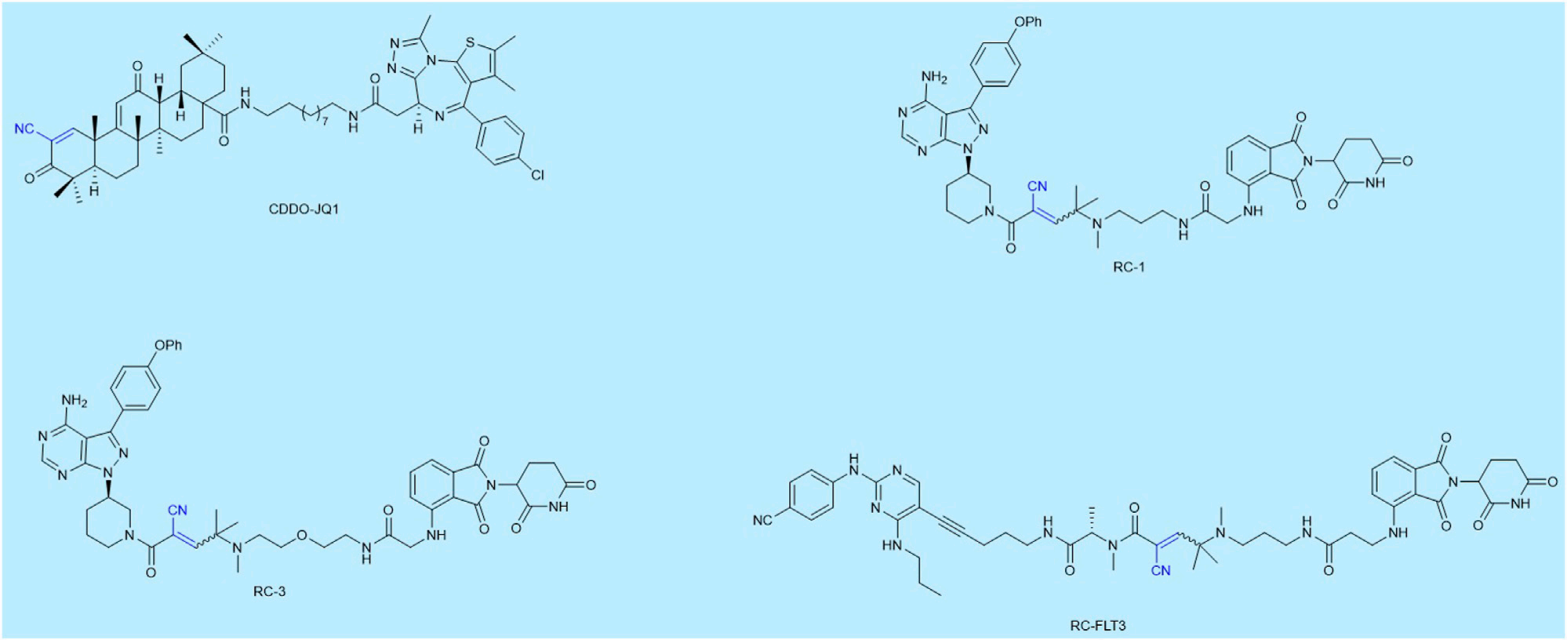
Examples of reversible covalent PROTACs. The names given to the molecules are taken from the publications in which they are described.

**TABLE 1:** Comparison of different types PROTACs with pros and cons.

Comparison of different types PROTACs with pros and cons						
Types	Name	Target	E3 ligase recruiters	DC_50_ (nM)	Pros	Cons
	CDDO-JQ1 Bingqi Tong et al. (2020)	BRD4	Bardoxolone	100–200	The first reversible covalent E3 ligase recruiter	Loss of BRD4 degradation at higher concentrations due to the “hook” effect Tong et al. (2020).
Reversible covalent PROTACs	RC-1 Guo et al. (2020b)	BTK	Pomalidomide	6.6	Enhance cellular uptake and target selectivity	—
	RC-3 Gabizon et al. (2020c)	BTK	Thalidomide	6	Exploit abundant biological nucleophiles as transport vehicles	—
Irreversible covalent PROTACs	IRC-1 Guo et al. (2020a)	BTK	Pomalidomide	—	—	Induce inefficient BTK degradation
	IR-1 Gabizon et al. (2020c)	BTK	Thalidomide	8.9	—	Poor selectivity and potential toxicity
Reversible noncovalent PROTACs	RNC-1 Guo et al. (2020a)	BTK	Pomalidomide	—	Better degradation efficiency	Poor selectivity
	NC-1 Gabizon et al. (2020c)	BTK	Thalidomide	2.2	Better degradation efficiency	Poor selectivity

In conclusion, reversible covalent PROTACs present a very promising and powerful approach for current drug discovery and tool development in biology with better selectivity, degradation activity, and longer duration of action compared to noncovalent and irreversible covalent PROTACs. Reversible covalent PROTACs can overcome the drawbacks of the other two types by avoiding a permanent protein complex and maintaining the catalytic nature of PROTACs. Currently, keys to designing a reversible covalent PROTAC are to discover a reversible covalent E3 recruiter or introduce a reversible covalent ligand binding to the target protein (KielyCollins et al., 2021), such as cyanoacrylamide (Guo W. H. et al., 2020) and dimethylated cyanoacrylamide (Gabizon et al., 2020b). On one hand, more target binders and E3 ligases applicable in the development of PROTACs are awaiting to be discovered. On the other hand, extra efforts are required to gain deeper insight into the clinical effectiveness and safety of PROTACs.

## Data Availability

The original contributions presented in the study are included in the article/Supplementary Material; further inquiries can be directed to the corresponding author.
